# Effects of dietary *Galla Chinensis* tannin supplementation on immune function and liver health in broiler chickens challenged with lipopolysaccharide

**DOI:** 10.3389/fvets.2023.1126911

**Published:** 2023-02-14

**Authors:** Peng Yuan, Haitao Xu, Yuanfei Ma, Jiaxing Niu, Yang Liu, Libo Huang, Shuzhen Jiang, Ning Jiao, Xuejun Yuan, Weiren Yang, Yang Li

**Affiliations:** ^1^Key Laboratory of Efficient Utilization of Non-grain Feed Resources (Co-construction by Ministry and Province), Ministry of Agriculture and Rural Affairs, Shandong Provincial Key Laboratory of Animal Biotechnology and Disease Control and Prevention, Department of Animal Science and Veterinary Medicine, Shandong Agricultural University, Tai'an, China; ^2^Animal Husbandry Development Center of Changyi City, Weifang, China; ^3^Agricultural and Rural Comprehensive Service Center of Bincheng District, Binzhou, China; ^4^College of Life Sciences, Shandong Agricultural University, Tai'an, China

**Keywords:** *Galla Chinensis*, tannic acid, broiler, liver health, lipopolysaccharide

## Abstract

Herein, *Galla Chinensis* tannin (GCT) was examined for its influence on preventing lipopolysaccharide (LPS)-induced liver damage in broiler chickens. Approximately 486 one-day-old healthy broilers were randomly allocated to 3 treatment groups (control, LPS, and LPS + GCT). The control and LPS groups were fed a basal diet and the LPS+GCT group was fed the basal diet supplemented with 300 mg/kg GCT. LPS was intraperitoneally injected (1 mg/kg body weight BW) in broilers in the LPS and LPS+GCT groups at 17, 19, and 21 days of age. The results manifested that dietary GCT addition attenuated LPS-induced deleterious effects on serum parameters and significantly increased serum immunoglobulin and complement C3 concentrations relative to the control and LPS groups. Dietary supplementation of GCT inhibited LPS-induced increase in broiler hepatic inflammatory cytokines, caspases activities, and TLR4/NF-κB pathway-related gene mRNA expression. Therefore, 300 mg/kg GCT addition to the diet improved the immune function of broilers and inhibit liver inflammation by blocking the *TLR4/NF-*κ*B* pathway. Our findings provide support for the application of GCT in poultry production.

## Introduction

The liver performs a multitude of functions, including immune activity, detoxification, phagocytizing bacteria, and nutrient metabolism. Its health is pertinent and indispensable to livestock production (1, 2). Contemporarily, in the poultry industry, intensive production has resulted in their confinement to limited spaces, exacerbating the risk of microbial infection. Substances absorbed from the gut are transported to the liver *via* the portal vein, making it the first barrier against enteric pathogenic microorganisms and their by-products (3–5). Liver inflammatory injury is a common clinical disease due to insensitive poultry production (6–8).

Tannins are naturally occurring polyphenolic compounds found in high concentrations in pasture, shrub, grain, herbal, and fruit species (9, 10). Depending on the chemical structure, tannins can be divided into hydrolyzable tannins (HT) and concentrated tannins (CT) (11). Tannins have exerted positive effects on growth performance, gut morphology, and antioxidant activity in broilers under normal and stressful conditions in previous studies (12–15). *Galla Chinensis* has long been used as a traditional medicine in China, and its main bioactive component is HT, gallotannins (16). Recently, 300 mg/kg *Galla Chinensis* tannin (GCT) addition in the broiler diet was shown to improve growth performance and immune functions (17). Moreover, Zhang et al. (18) also showed that 250 and 500 mg/kg GC improved growth performance and antioxidant capacity, along with protecting gut health in AFB1-challenged broilers to an extent. Whether supplementing GCT in the diet can alleviate liver injury under inflammatory conditions in broiler chickens remains unknown.

As a by-product of microbes, lipopolysaccharide (LPS) is a cell wall component of Gram-negative bacteria. LPS activates acute immune responses and induces systemic dysfunction *via* generating proinflammatory cytokines that can inhibit innate immune receptors expressions and downregulate immunoglobulin synthesis (19). Upon recognition of LPS by the liver immune system, cytokines are generated, which in turn result in liver inflammation (20, 21). Huang et al. (22) have indicated that intraperitoneal injection of LPS can cause acute liver injury and alter hepatic structure and function by regulating toll-like receptor 4 (TLR4) signaling and its downstream molecules along with specific cytokines in broilers.

Therefore, this study aimed to investigate the effect of adding 300 mg/kg GCT in the diet on alleviating systemic and liver inflammatory responses in broiler chickens challenged by LPS. Our findings provide useful insights for the application of GCT in poultry production.

## Materials and methods

### Experimental design and management

A total of 486 1-day-old Arbor Acres (AA) broiler chickens with uniform body weight (48.35 ± 0.41g) were chosen and randomly allocated to 3 treatment groups, each of which included 6 replicates per treatment (27 chickens in each replicate), for a 21-day study. The three treatment groups were control (CON), LPS, and LPS+GCT. Broilers in CON and LPS groups were fed a basal diet and those in the LPS+GCT group were fed a basal diet supplemented with 300 mg/kg GCT (17). The LPS (*E. coli* L2880; Sigma-Aldrich, MO, USA) was intraperitoneally injected (1 mg/kg body weight BW) in broilers in the LPS and LPS+GCT groups at 17, 19, and 21 days of age, while an equivalent amount of sterile saline solution was injected in the CON group intraperitoneally (23). The basal diet (Table 1) was formulated to provide nutrients that met or exceeded the needs of the broilers as recommended by the National Research Council (24). Microencapsulated GCT products with a net 40% TA content were produced by Wufeng Chicheng Biotechnology Co., Ltd., Yichang, Hubei, China. During the experimental period, all chickens were raised in standard chicken coops with free access to water and food. The test site and chicken house were cleaned and sanitized daily, and normal disinfection was conducted. The test house was well-ventilated and maintained at the desired temperature. Strict anti-epidemic and disinfection measures were implemented during the trial. On the 7th and 14th days of the experiment, the birds were vaccinated with the Newcastle disease vaccine and infectious bursal disease vaccine, respectively.

**Table 1:** Composition and nutrient contents of the basal diets (air-dried basis).

**Items**	**Content**
Ingredients, %	
Corn	55.91
Soybean meal, 44% CP	13.78
Wheat bran	11.98
Corn starch residue	7.99
Corn gluten meal	3.99
Extruded soybean	1.50
Limestone	1.70
Calcium monophosphate	1.10
L-Lysine HCl	1.00
DL-Methionine	0.20
L-Threonine	0.10
Sodium chloride	0.40
Choline	0.10
Phytase	0.10
Complex enzyme	0.02
Trace mineral premix^1^	0.10
Vitamin premix^2^	0.02
Antioxidant	0.02
Total	100
Calculated analysis,%	
Metabolizable energy MJ/kg	12.33
Crude protein	19.47
Crude fat	3.45
Calcium	0.94
Available phosphorus	0.35
Lysine	1.15
Methionine	0.50

^1^Provided per kilogram of the complete basal diets: 10 mg of Cu as CuSO_4_, 100 mg of Fe as FeSO_4_, 1.1 mg of I as Ca(IO_3_)_2_, 65 mg of Zn as ZnSO_4_, 100 mg of Mn as MnSO_4_ and 0.3 mg of Se as Na_2_SeO_3_.

^2^Provided per kilogram of complete basal diet: vitamin A 10,000 IU, vitamin D_3_ 3,000 IU, vitamin E 30 IU, vitamin K_3_ 1.3 mg, vitamin B_1_ 2.2 mg, vitamin B_2_ 8 mg, vitamin B_3_ 8 mg, vitamin B_6_ 4 mg, vitamin B_12_ 0.025 mg, biotin 0.2 mg, niacin 40 mg, folic acid 1 mg and D-calcium pantothenate 10 mg.

### Samples collection

Three hours after LPS or saline solution injection at 21 days of age (23), one broiler in each replicate was chosen and 5 mL of wing vein blood samples were collected using a coagulation vacuum container tube. The sera were obtained by centrifugation at 4,000 × *g* for 15 min at 4°C and stored at −80°C until further analysis. Subsequently, the broilers were euthanized and slaughtered for collecting liver samples. Initially, a part of the liver samples was quickly frozen with liquid nitrogen and stored at −80°C until further analysis, while another portion was fixed for 24 h in a 4% paraformaldehyde solution.

### Determination of serum biochemical parameters and inflammatory cytokines concentrations

The commercial kits (Jiancheng Bioengineering Institute, Nanjing, China) were used to detect alanine aminotransferase (ALT), total protein (TP), low-density lipoprotein (LDL), albumin (ALB), total cholesterol (TCHO), urea nitrogen (UREA), triglyceride (TG), high-density lipoprotein (HDL), and glucose (GLU) concentrations in the sera on the Roche Cobus-Mira-Plus platform (Roche Diagnostic System Inc., USA) (25). The levels of inflammatory cytokines including tumor necrosis factor α (TNF-α), interleukin (IL)-1β, IL-4, IL-6, and IL-10 were examined using chicken-specific ELISA kits (Jiangsu Meimian Industrial Co., Ltd, Jiangsu, China) and all experimental steps were conducted following the corresponding protocols described by Li et al. (23).

### Determination of serum immunoglobulins and complements concentrations

ELISA kits (Jiangsu Meimian) were used to detect serum immunoglobulin (Ig)G, IgM, IgA, complement 3 (C3), and complement 4 (C4) levels following the detection procedures described previously (26).

### Examination of hepatic histopathology

Liver samples were fixed in a 4% paraformaldehyde solution for 24 h, dehydrated with graded concentrations of ethyl alcohol, and embedded in liquid paraffin according to the standard histological procedure (17). After embedding the liver tissue in paraffin wax, 5 μm sections were cut and stained with hematoxylin and eosin (H&E). An Olympus digital microscope (Olympus BX51, Tokyo, Japan) was used to observe the liver sections.

### Determination of inflammatory cytokines, health biomarkers and caspases levels in liver

ELISA kits (Jiangsu Meimian) were used to detect the levels of TNF-α, IL-1β, IL-6, IL-18, NOD-like receptor family pyrin domain containing 3 (NLRP3), heat shock 70 kDa protein (HSP70), caspase-1, caspase-3, and hepatic 8-hydroxy-2-deoxyguanosine(8-OHdG) following the corresponding protocols described by Chen et al. (27).

### Determination of gene expression

Following the manufacturer's instructions, total RNA was extracted from the liver samples using the TRIzol reagent (Invitrogen, Carlsbad, CA, USA). Subsequently, cDNA was synthesized using TaKaRa reverse transcription kit (TaKaRa, Dalian, China), and amplified by real-time quantitative polymerase chain reaction (RT-PCR) using the specific kit (TaKaRa, Dalian, China) following the protocol described by Li et al. (28). All primer sequences (Table 2) were based on the articles of Niu et al. (17), including those for TLR4, nuclear factor-kappa B (NF-κB), myeloid differentiation primary response 88 (MyD88), NLRP3, B-cell lymphoma-2 (Bcl-2), and Bcl-2-associated X (Bax), were synthesized by Sangon Bioengineering Ltd. (Shanghai, China). The 2^−ΔΔ^CT method was used to evaluate the target mRNA expression relative to the levels of the internal reference gene, β-actin.

**Table 2:** Primer sequences used for quantitative real-time PCR.

**Genes**	**Gene bank No**.	**Primer sequences^a^ (5'-3')**	**Size, bp**
*β-actin*	NM_205518.1	F: TTGGTTTGTCAAGCAAGCGG	100
		R: CCCCCACATACTGGCACTTT	
*TLR4*	NM_001030693.1	F: AGGCACCTGAGCTTTTCCTC	96
		R: TACCAACGTGAGGTTGAGCC	
*MyD88*	XM_046910878.1	F: TGATGCCTTCATCTGCTACTG	174
		R: TCCCTCCGACACCTTCTTTCTA	
*NF-κB*	NM_001396038.1	F: CAGCCCATCTATGACAACCG	152
			R: TCAGCCCAGAAACGAACCTC
*NLRP3*	NM_001348947.2	F: GAAGGTGCTGCTATGGACATTG	118
			R: CGTGCTCTGTGTATTTCTGCTTAT
*Bax*	XM_422067	F: GGTGACAGGGATCGTCACAG	108
			R: TAGGCCAGGAACAGGGTGAAG
*Bcl-2*	NM_205339.2	F: GCTGCTTTACTCTTGGGGGT	128
			R: CTTCAGCACTATCTCGCGGT

^a^F, forward; R, reverse.

### Statistical analysis

After the data were evaluated for normality by the Shapiro-Wilk's statistic (*W* > 0.05), one-way analysis of variance (ANOVA) with the SAS software (Version 9.4, Institute Inc., Cary, NC, US) was used to assess statistically significant differences among the three treatments. Multiple comparison analyses were performed by the least significant difference procedure. Data were presented using the mean ± standard error and plotted. Significant differences were considered at ^*^p < 0.05, ^**^p < 0.01, and ^***^p < 0.001, and ^#^0.05 < *p* < 0.10 was regarded as a trend toward significance. “ns” represented non-significant differences.

## Results

### Effects of GCT supplementation on serum concentrations of biochemical parameters

**Figure 1:**
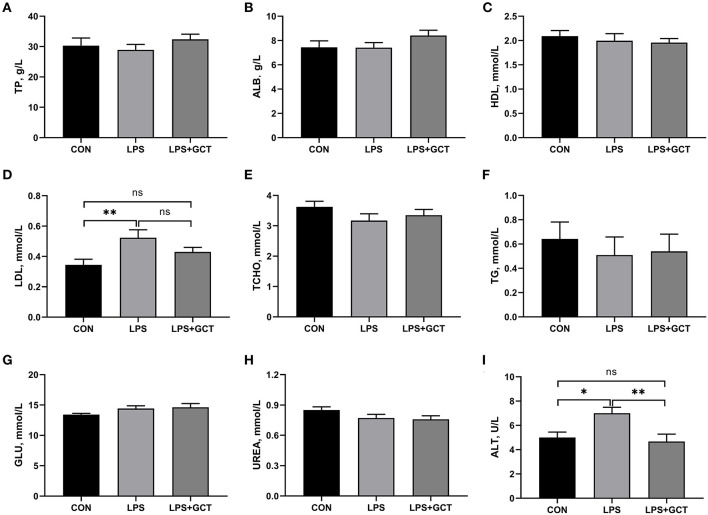
Effects of dietary *Galla Chinensis* tannin (GCT) supplementation on serum biochemical parameters of broilers under lipopolysaccharide (LPS) challenge. **(A)** Total protein (TP); **(B)** Albumin (ALB); **(C)** High-density lipoprotein (HDL); **(D)** Low-density lipoprotein (LDL); **(E)** Total-cholesterol (TCHO); **(F)** Triglyceride (TG); **(G)** Glucose (GLU); **(H)** Urea nitrogen (UREA); **(I)** Alanine transaminase (ALT). CON, fed on a basal diet and intraperitoneally injected with saline solution; LPS, fed on a basal diet and intraperitoneally injected with LPS; LPS+GCT, fed on a basal diet supplemented with 300 mg/kg GCT and intraperitoneally injected with LPS. Values are presented as mean ± standard error (*n* = 6). *p < 0.05, **p < 0.01; ns, non-significant.

As displayed in Figure 1, compared to CON broilers, LPS broilers showed significantly higher serum LDL and ALT levels (*p* < 0.05), and 300 mg/kg GCT supplementation suppressed the LPS-induced increase in serum LDL and ALT levels to those observed in the CON group (*p* < 0.05). However, there were no significant differences in serum TP, ALB, HDL, TCHO, TG, GLU, and UREA contents in broilers among the three treatment groups (*p* >0.05).

### Effects of GCT supplementation on serum inflammatory cytokines concentrations

**Figure 2:**
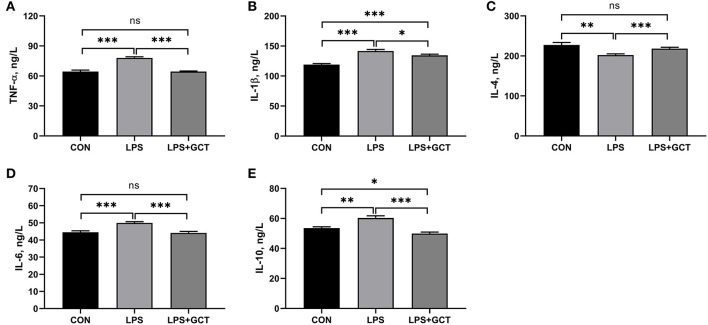
Effects of dietary *Galla Chinensis* tannin (GCT) supplementation on serum inflammatory factors of broilers under lipopolysaccharide (LPS)-challenge. **(A)** Tumor necrosis factor-α (TNF-α); **(B)** Interleukin-1β (IL-1β); **(C)** Interleukin-4 (IL-4); **(D)** Interleukin-6 (IL-6); **(E)** Interleukin-10 (IL-10). CON, fed on a basal diet and intraperitoneally injected with saline solution; LPS, fed on a basal diet and intraperitoneally injected with LPS; LPS+GCT, fed on a basal diet supplemented with 300 mg/kg GCT and intraperitoneally injected with LPS. Values are presented as mean ± standard error (*n* = 6). *p < 0.05, **p < 0.01, ***p < 0.001; ns, non-significant.

Significantly enhanced serum TNF-α, IL-6, IL-1β, and IL-10 concentrations and decreased serum IL-4 levels were observed in LPS broilers relative to CON broilers (*p* < 0.05; Figure 2). The addition of GCT significantly reduced serum TNF-α, IL-1β, IL-6, and IL-10 concentrations and increased that of IL-4 in the LPS-challenged broilers (*p* < 0.05). The CON group showed significantly lower IL-1β level and significantly higher IL-10 concentration in serum than the LPS+GCT group (*p* < 0.05). However, there were no significant differences in serum TNF-α, IL-4, and IL-6 concentrations between CON and LPS+GCT groups (*p* > 0.05).

### Effects of GCT supplementation on serum levels of immunoglobulins and complements

**Figure 3:**
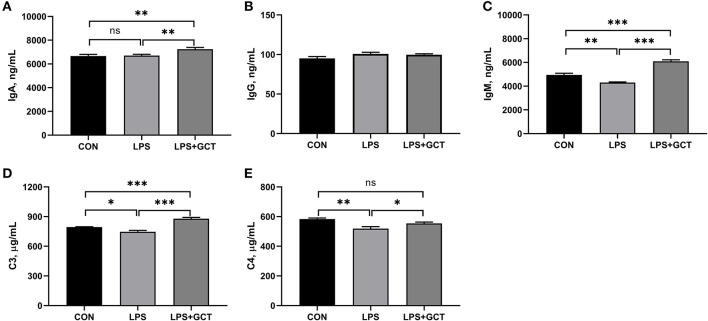
Effects of dietary *Galla Chinensis* tannin (GCT) supplementation on serum immunoglobulins and complements of broiler chickens under lipopolysaccharide (LPS)- challenge. **(A)** Immunoglobulin A (IgA); **(B)** Immunoglobulin G (IgG); **(C)** Immunoglobulin M (IgM); **(D)** Complement C3; **(E)** Complement C4. CON, fed on a basal diet and intraperitoneally injected with saline solution; LPS, fed on a basal diet and intraperitoneally injected with LPS; LPS+GCT, fed on a basal diet supplemented with 300 mg/kg GCT and intraperitoneally injected with LPS. Values are presented as mean ± standard error (*n* = 6). *p < 0.05, **p < 0.01, ***p < 0.001; ns, non-significant.

As displayed in Figure 3, significantly reduced serum IgM, complement C3, complement C4 concentrations were observed in LPS broilers, and GCT supplementation significantly increased serum IgA, IgM, complement C3, and complement C4 concentrations in LPS-challenged broilers (*p* < 0.05). Besides, the LPS+GCT broilers showed significantly higher serum IgA, IgM, and complement C3 concentrations than the CON broilers (*p* < 0.05), and there was no significant difference in serum complement C4 between broilers in CON and LPS+GCT groups (*p* > 0.05).

### Effects of GCT supplementation on hepatic histopathology

**Figure 4:**
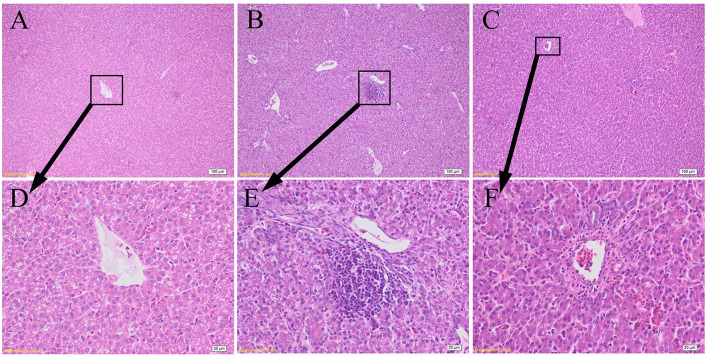
Effects of dietary *Galla Chinensis* tannin (GCT) supplementation on hepatic histopathology. **(A–C)** Hematoxylin and eosin photomicrographs of CON, LPS, and LPS+GCT obtained at 100× magnification, respectively; **(D–F)** Hematoxylin and eosin photomicrographs of CON, LPS, and LPS+GCT obtained at 400× magnification, respectively. CON, fed on a basal diet and intraperitoneally injected with saline solution; LPS, fed on a basal diet and intraperitoneally injected with LPS; LPS+GCT, fed on a basal diet supplemented with 300 mg/kg GCT and intraperitoneally injected with LPS.

As shown in Figure 4, the hepatocytes in CON group were structurally complete and arranged in an orderly manner with minimal hyperchromatism, and showed normal development hepatic sinusoid but macrovesicular fatty change. The hepatocytes in LPS group showed obvious injury, including dissolved nucleus, cellular degeneration, and inflammatory cell infiltration with few lymphocytes. The hepatocytes in LPS+GCT group were structurally complete and arranged in an orderly manner with hyperchromatism, and also showed normal development hepatic sinusoid. However, LPS+GCT group showed fewer inflammatory cell infiltration and obvious cell divide and repair compared with LPS group.

### Effects of GCT supplementation on hepatic levels of inflammatory cytokines and caspases

**Figure 5:**
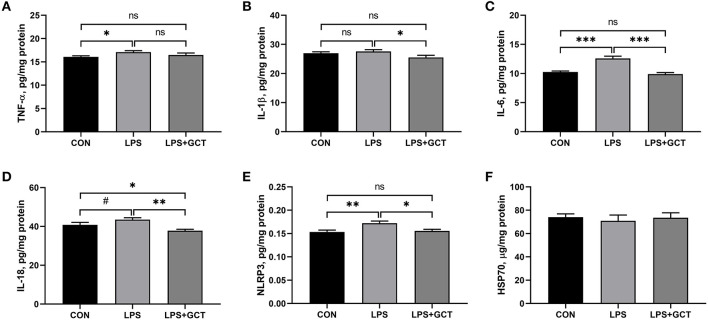
Effects of dietary *Galla Chinensis* tannin (GCT) supplementation on hepatic inflammatory cytokines of broilers under lipopolysaccharide (LPS)-challenge. **(A)** Tumor necrosis factor-α (TNF-α); **(B)** Interleukin-1β (IL-1β); **(C)** Interleukin-6 (IL-6); **(D)** Interleukin-18 (IL-18); **(E)** NLRs family pyrin domain containing 3 (NLRP3); **(F)** Heat shock 70 kDa protein (HSP70). CON, fed on a basal diet and intraperitoneally injected with saline solution; LPS, fed on a basal diet and intraperitoneally injected with LPS; LPS+GCT, fed on a basal diet supplemented with 300 mg/kg GCT and intraperitoneally injected with LPS. Values are presented as mean ± standard error (*n* = 6). ^*^p < 0.05, ^**^p < 0.01, ^***^p < 0.001, ^#^0.05 < *p* < 0.10; ns, non-significant.

As displayed in Figure 5, compared to the CON broilers, the LPS broilers showed markedly higher TNF-α, IL-6, and NLRP3 levels (*p* < 0.05), and tended to increase IL-18 level (*p* < 0.10) in their livers. However, 300 mg/kg GCT supplementation suppressed the LPS-induced raising in the hepatic levels of TNF-α, IL-6, and NLRP3 to those observed in the CON group (*p* < 0.05). Moreover, the concentrations of IL-1β and IL-18 in the LPS+GCT group were markedly lower than those in the LPS group (*p* < 0.05), and IL-18 level was notably lower in the LPS+GCT group than in the CON group (*p* < 0.05).

### Effects of GCT supplementation on the hepatic caspases and 8-OHDG levels

**Figure 6:**
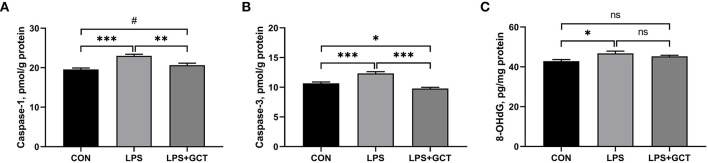
Effects of dietary *Galla Chinensis* tannin (GCT) supplementation on hepatic caspases levels of broilers under lipopolysaccharide (LPS)-challenge. **(A)** Caspase-1; **(B)** Caspase-3; **(C)** 8-hydroxy-2-deoxyguanosine (8-OHdG). CON, fed on a basal diet and intraperitoneally injected with saline solution; LPS, fed on a basal diet and intraperitoneally injected with LPS; LPS+GCT, fed on a basal diet supplemented with 300 mg/kg GCT and intraperitoneally injected with LPS. Values are presented as mean ± standard error (*n* = 6). ^*^p < 0.05, ^**^p < 0.01, ^***^p < 0.001, ^#^0.05 < *p* < 0.10; ns, non-significant.

Hepatic caspases and 8-OHDG levels are displayed in Figure 6. Compared to the CON group, LPS administration markedly elevated the caspase-1, caspase-3, and 8-OHDG levels in the livers of broilers (*p* < 0.05). Dietary 300 mg/kg GCT addition significantly decreased hepatic caspase-1 and caspase-3 activities in the liver compared to the LPS broilers (*p* < 0.05). Moreover, the LPS+GCT broilers showed significantly lower caspase-3 activity (*p* < 0.05), and tended to have higher caspase-1 activity in the liver compared to the CON broilers (*p* < 0.10).

### Effects of GCT supplementation on hepatic genes expressions

**Figure 7:**
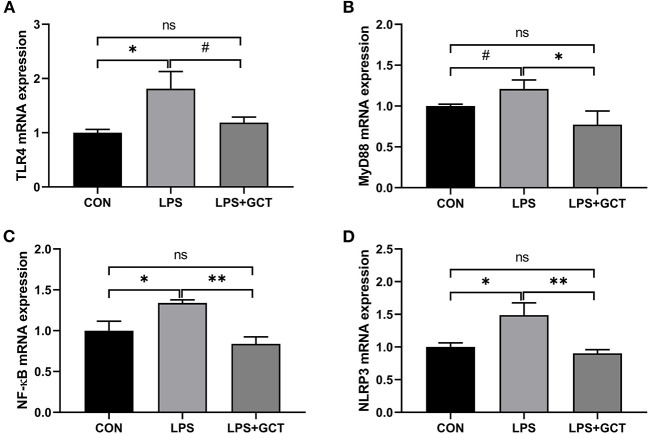
Effects of dietary *Galla Chinensis* tannin (GCT) supplementation on expressions of inflammatory pathway genes in liver of broilers under lipopolysaccharide (LPS)-challenge. **(A)** Toll-like Receptor 4 (*TLR4*); **(B)** Myeloid differentiation primary response 88 (*MyD88*); **(C)** Nuclear factor-kappa B (*NF-*κ*B*); **(D)** NLRs family pyrin domain containing 3 (*NLRP3*). CON, fed on a basal diet and intraperitoneally injected with saline solution; LPS, fed on a basal diet and intraperitoneally injected with LPS; LPS+GCT, fed on a basal diet supplemented with 300 mg/kg GCT and intraperitoneally injected with LPS. Values are presented as mean ± standard error (*n* = 6). *p < 0.05, **p < 0.01, ^#^0.05 < *p* < 0.10; ns, non-significant.

Inflammatory pathway gene expressions in the liver are displayed in Figure 7. Compared to the CON broilers, the LPS broilers showed significantly up-regulated mRNA expressions of *TLR4, NF-*κ*B*, and *NLRP3* (*p* < 0.05), and tended to have an increased *MyD88* mRNA expression (*p* < 0.10) in the liver. However, 300 mg/kg GCT supplementation suppressed LPS-induced increase in hepatic *MyD88, NF-*κ*B*, and *NLRP3* mRNA expressions to those observed in the CON group (*p* < 0.05). Moreover, hepatic *TLR4* expression of LPS+GCT broilers tended to be lower than that of LPS broilers and showed no statistically significant difference compared with the CON broilers (*p* > 0.05).

**Figure 8:**
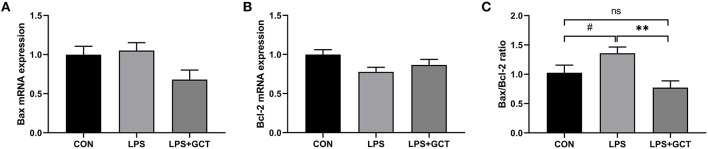
Effects of dietary *Galla Chinensis* tannin (GCT) supplementation on apoptosis genes in livers of broilers under lipopolysaccharide (LPS)-challenge. **(A)** BCL2-Associated X (*Bax*); **(B)** B-cell lymphoma-2 (*Bcl-2*); **(C)** *Bax/Bcl-2* ratio. CON, fed on a basal diet and intraperitoneally injected with saline solution; LPS, fed on a basal diet and intraperitoneally injected with LPS; LPS+GCT, fed on a basal diet supplemented with 300 mg/kg GCT and intraperitoneally injected with LPS. Values are presented as mean ± standard error (*n* = 6). **p < 0.01, ^#^0.05 < *p* < 0.10; ns, non-significant.

Hepatic apoptosis-related gene expressions are shown in Figure 8. No significant differences were observed in mRNA expression of pro-apoptosis *Bax* and anti-apoptosis *Bcl-2* among all the groups (*p* > 0.05). However, *Bax*/*Bcl-2* ratio in the LPS group tended to be higher than that in the CON group (*p* < 0.10), and was markedly higher than that in the LPS+GCT (*p* < 0.05) group; there was no significant difference in *Bax*/*Bcl-2* ratio between the CON group and LPS+GCT group *(p* > 0.05).

## Discussion

Previous studies have shown that LPS injection can cause liver injury and dysfunction in broilers (22). When a liver injury occurs, ALT in hepatocytes enters the blood circulation, increasing serum ALT levels (29). Therefore, ALT is usually regarded as a specific indicator of liver injury in clinical settings (30). In the present study, the results showed that the ALT activity of the GCT group was similar to the serum of broilers in the CON group but significantly higher in the LPS group. The liver is one of the main sites of complement synthesis. Complement is an enzymatically active glycoprotein that plays a key role in the body's immune regulation, and liver damage is usually associated with decreased serum levels of complements (31). Complement C3 deficiency results in decreased liver regeneration (32). We found that GCT supplementation significantly increased serum C3 concentration compared to the CON and LPS groups, and inhibited the decrease in serum C4 concentration induced by the LPS challenge. LPS administration significantly increased hepatic 8-OHdG concentration compared to the CON group. 8-OHdG is considered as the most critical indicator of DNA damage because it can result in the formation of 8-OHdG in the nucleotide pool during DNA replication (33). The above results demonstrated that the animal model of LPS-induced liver injury was successfully established, as evidenced by the hepatic histopathology. Supplementation with GCT in the diet suppressed the adverse changes in serum ALT and complement levels along with hepatic 8-OHdG levels, showing that GCT supplementation benefited the amelioration of LPS-induced liver injury in broilers.

Immune function decline is associated with the development of liver diseases (34). In modern breeding, intensive farming systems cause drastic metabolism in broiler chickens during growth and development, resulting in decreased immune function (25). Our findings showed that the LPS challenge decreased serum IgM concentration but GCT addition increased serum IgA and IgM concentrations in these broilers compared to the other groups. Immunoglobulin acts as a major anti-infective component in the blood and serves as the first line of host defense against infections (35). Previous study in IPEC-J2 cells also indicated that LPS decreased IgA, IgG, and IgM levels (36). Niu et al. (17) showed that GCT addition in the broiler diet elevated the serum levels of IgG, IgM, and complements. Therefore, enhanced immune function by GCT supplementation might contribute to alleviating LPS-induced liver injury.

Increasing evidence shows that inflammatory response is a major important inducement for liver injury (37). LPS challenge can increase liver contents of pro-inflammatory cytokines like TNF-α, IL-1β, and IL-6, and result in inflammatory injuries to broilers (22, 38). In the present study, LPS administration induced an increase in the concentrations of TNF-α, IL-6, IL-1β, and IL-10, while reducing the serum IL-4 content. Moreover, the concentrations of TNF-α, IL-6, and IL-18 in the liver were elevated. TNF-α, IL-1β, IL-6, and IL-18 are often used as markers of inflammatory responses and are involved in the induction of liver diseases (39, 40). The main biological roles of IL-4 and IL-10 are inhibition of the production and release of pro-inflammatory cytokines, promoting the proliferation and differentiation of B cells, and enhancing the activity of macrophages (41, 42). However, IL-10 production can be also increased under low-grade inflammatory conditions for inducing antibody generation (26). Therefore, the LPS challenge resulted in a systemic and hepatic inflammatory response in this study. Our findings showed that GCT addition suppressed LPS-induced detrimental changes in inflammatory cytokines concentrations. A previous study *in vitro* also indicated that tannin addition could inhibit the production of pro-inflammatory cytokines, IL-1β and IL-6, in BV2 microglial cells (43). *In vivo* experiments in mice have shown that tannic acid promotes the reduction in TNF-α, IL-1β, and IL-6 contents (44). Furthermore, caspases play a key role in mediating inflammatory responses and cellular apoptosis (45). Dietary GCT supplementation inhibits LPS-induced increase in hepatic caspase-1 and caspase-3 activities of broilers. Caspase 1, an initiating caspase or the IL-1β converting enzyme, is required for apoptosis (46). Caspase-3 is an executioner caspase that causes apoptosis-related nuclear changes, including chromatin condensation (47). A high Bax/Bcl-2 ratio usually results in the activation of caspase-1 and caspase-3 in initiating pyroptosis and apoptosis, respectively, ultimately leading to organ damage (48). In this study, dietary GCT addition alleviated the LPS-induced increases in the Bax/Bcl-2 ratio. Importantly, dietary GCT could resist LPS-induced liver injury by inhibiting systemic and hepatic inflammatory responses.

To further investigate the possible mechanisms underlying the inhibition of LPS-induced broiler liver inflammatory injury by GCT, the gene expression levels of the TLR4/NF-κB pathway were quantified herein. TLR4, belonging to the pattern recognition receptor family of genes, plays an important role in activating the innate immune response, participating in the body's response to infection due to various pathogens (49). TLR4 can quickly trigger the activation and generation of several cytokines involved in regulating immunological and inflammatory responses by activating the NF-κB signaling pathway through the MyD88 protein (50, 51). Previous studies have shown that intraperitoneal injection of LPS can mediate acute liver injury by up-regulating TLR4 and its downstream molecules including MyD88, NF-κB, IL-1β, and TNF-α (22). Gastric administration of 20 and 40 mg/kg of pure tannic acid down-regulates the expression of NF-κB, IL-6, and TNF-α induced by arsenic trioxide in rats (52). In the present study, the LPS challenge up-regulated the expressions of TLR4/NF-κB pathway-related genes and dietary GCT supplementation alleviated LPS-induced increase in the hepatic mRNA expressions of TLR4, MyD88, NF-κB, and NLRP3. Taken together, GCT could suppress hepatic inflammatory responses likely by inhibiting the TLR4/NF-κB signaling pathway.

## Conclusion

To sum up, our findings demonstrated that addition with 300 mg/kg GCT could alleviate the systemic and hepatic inflammatory responses in broilers challenged by LPS. These findings can facilitate the mechanistic understanding by which dietary GCT can promote liver development and enhance immune regulation, supporting the application of GCT in poultry production.

## Data availability statement

The original contributions presented in the study are included in the article/supplementary material, further inquiries can be directed to the corresponding authors.

## Ethics statement

The animal study was reviewed and approved by the Animal Care and Use Committee of Shandong Agricultural University. The protocol for this experiment involving animals was in accordance with the guidelines of the Animal Care and Use Committee of Shandong Agricultural University (Ethics approval code: SDAUA-2021-019).

## Author contributions

Conceptualization: PY, WY, and YLi. Methodology, data curation, and project administration: PY, JN, and YLiu. Software: WY, HX, YM, LH, and YLi. Validation, visualization, and supervision: LH and YLi. Formal analysis: PY, HX, YM, and LH. Investigation and resources: NJ, SJ, and XY. Writing—original draft preparation: PY. Writing—review and editing: YLi. Funding acquisition: WY and YLi. All authors have read and agreed to the published version of the manuscript.
